# Strengthening Africa’s capacity for vaccine research: Needs and challenges

**DOI:** 10.34172/hpp.2022.36

**Published:** 2022-12-10

**Authors:** Adedoyin John-Joy Owolade, Taiwo Oluwaseun Sokunbi, Favour Oluwatobi Aremu, Esther Oluwatosin Omotosho, Blessing Abai Sunday, Yusuff Adebayo Adebisi, Aniekan Ekpenyong, Abdulhammed Opeyemi Babatunde

**Affiliations:** ^1^Faculty of Pharmacy, Obafemi Awolowo University, Ile Ife, Nigeria; ^2^Faculty of Pharmacy, University of Ibadan, Ibadan, Nigeria; ^3^Faculty of Pharmacy, University of Uyo, Uyo, Nigeria; ^4^Global Health Policy Unit, University of Edinburgh, Scotland, United Kingdom; ^5^Department of Medicine and Surgery, Faculty of Clinical Sciences, College of Medicine, University of Ibadan, Ibadan, Oyo State, Nigeria

**Keywords:** Africa, Research, Communicable diseases, Vaccination, Vaccines

## Abstract

Vaccine development and production harbinger the control and eradication of infectious diseases. Vaccination played a huge role in the curtailment of disease outbreaks like smallpox and polio, especially in Africa. Despite the high demand for several vaccines in Africa due to the highly infectious disease burden, the continent still lacks adequate capacity for vaccine research and development. This paper aims to discuss the need and challenges of Africa to strengthen its capacity for vaccine research and development and also highlight practical recommendations. Some of the needs for Africa to prioritize vaccine research and development include; improving quality of life and well-being, cost-effectiveness, independent preparedness and response to local outbreaks, and increased access to funding. Challenges associated with vaccine research and development include the cost of the investment, risk of failure; poor ethical framework and legislation; lack of adequate funding; lack of political will & support; and poor surveillance system. Strategies to create sufficient research funds, an efficient surveillance system, and a legislative framework are clearly described. In conclusion, strengthening vaccine research capacity in Africa requires the political goodwill of African governments and strategic partnerships with international organizations and institutes. The challenges facing this development and possible solutions have been highlighted in this article.

## Introduction

 Vaccine development is crucial in the eradication of infectious diseases. Diseases such as rubella, measles, mumps, and smallpox have been successfully eradicated, while polio is also on the verge of being eradicated as a result of the development of their vaccines.^1^ These diseases accounted for the mortality of millions of individuals prior to the advent of vaccines. In August 2020, United Nations Children’s Fund (UNICEF) and the World Health Organization (WHO) congratulated Nigeria, the most populous country in Africa, on being declared poliovirus free, while recommending that continuous vaccination should be carried out on children less than five years of age.^2^ These feats were achievable because of the commendable energy and efforts toward vaccine research and development.^3^

 In a continent like Africa, the lack of adequate research funding, capacities, and environments, coupled with under-developed laboratories, have limited the capacity of scientists and researchers to engage in research and development.^4^ Despite the continent having the highest mortality rate of infectious diseases, universities and research institutes have remained grossly underfunded and ill-equipped to tackle pressing challenges.^4^ However, in recent years, visible progress has been recorded due to the availability of foreign funds and grants; the Fogarty International Center (FIC) spent about US$33 million on research ethics capacity development worldwide, with about US$10 million invested in Sub –Saharan Africa between 2000 and 2012.^5^ The European Developing Countries Clinical Trials Partnership (EDCTP) also invested over US$3 million in 54 research institutes in Africa within this period.^5^ It may be worthwhile to note that the seeming impediment to growth and capacity development in African research institutes may not necessarily be limited to funds, human resources, and infrastructures but also to the maintenance capacity and sustainability of its limited resources.^5^

 In this paper, we examine the need for Africa to strengthen its capacity for vaccine research, the likely challenges encountered in that quest, and finally, proffer recommendations on the way forward.

## Needs for strengthening Africa’s capacity for Vaccine research

 There is a need for the public sector to engage effectively in vaccine research and to help the improvement of newly discovered vaccines. Vaccination is one of the most cost-effective and efficient public health measures in curtailing disease, especially among children.^6^ It is an essential intervention at the crossroads between an individual’s choice and group immunity.^7^ To strengthen Africa’s capacity for vaccine research, investment for vaccine research could be triggered, and partnerships which include cooperation between Governments, charitable foundations, investment institutions, etc can be strategically established.^8^

 Aside from the need for vaccine research in Africa, the ineffectiveness of vaccination in some regions is enough to discourage investors from contributing to vaccine research and production. The current inflation and economic circumstances in many African nations are not encouraging. The COVID-19 effect and recurring inflation in African nations pose a significant barrier to investing in vaccine research. Most African countries will focus on strengthening their economy instead of investing in vaccine research and development.^9^

 Despite the availability of the AstraZeneca vaccine to tackle the global health crisis that kicks started in our continent on February 14, 2020 in Egypt,^10^ insufficient storage guidelines due to power outage stands as a barrier to proper handling and storage.^10^ For instance, South Sudan said it would discard 59 000 doses of the AstraZeneca vaccination, while Malawi destroyed over 20 000 doses. These vaccines, such as the AstraZeneca vaccine, require refrigerated conditions for up to six months.^9^ A lack of trained professionals has also been found to hold back vaccine rollout.^11^ It is of utmost importance that people’s perceptions and beliefs about vaccination are addressed if it would be effective in yielding the clinical health outcome desired. As much as this study aims to give clear introspects on vaccine research in Africa, underlying conditions cannot be disputed simultaneously. Change is evolution, and its resultant effects can start from vaccines through thorough research and a close look into its production in Africa.

## Challenges faced in the development of and Research for Vaccines

 A significant challenge with vaccine research and development in Africa is the investment required and the accompanying risk of failure.^12^ For instance, the development of simple vaccines could require an investment of about US$ 500 million, while complex vaccines could cost up to a billion dollars.^13^ These funds are not readily available to health systems in the region, which are already grappling with other significant challenges. However, global intervention programs have been established to speed up vaccine development for countries in the global south, such as the Global Alliance for Vaccines and Immunization (GAVI), the Bill Gates and Melinda Foundation Investments, and the World Health Organization Initiative for Vaccine Research amongst others. These organizations exist as to the sustainability and scalability of these interventions.^13^ In addition, most of these initiatives do not directly improve the research capacity (both human and infrastructure) of benefitting countries. However, a case is to be made of the Drugs for Neglected Diseases Initiative (DNDi), which was established to develop affordable treatments for neglected diseases, which are seldom worked on due to their unattractiveness to market incentified pharma or biotechnological industries. An important strategy to build partnerships with local scientists, researchers, governments, non-governmental organizations, other local partners, and pharmaceutical and biotechnological companies was adopted. Thus, translating to open and collaborative research and development.^14^

 In many sub-Saharan countries, “legislative framework” is not constantly evaluated to align with the new trends in research, which includes; rights associated with intellectual property, well-structured and approved conduct of clinical trials, including having sufficient research study protocols as well as other important factors, these gaps prevent the effective management of research activities and a conducive research environment needed.^15^ However, the WHO brought about initiatives to help African countries come together to discuss common challenges and seek how to achieve synchronized regulatory structures for clinical trial conduct. One of which was a workshop brought by the Department of Immunization, Vaccines and biologics in 2005, which involved 13 African countries coming together to review the legal framework of each country, and each country’s regulatory procedures and this birthed a platform called the African Vaccine Regulatory Forum (AVAREF) since 2006. The goal of the Forum would be to build member countries’ capacity for adequate regulation of clinical trials, identify lapses and jointly seek support from the WHO. However, despite these interventions by the WHO, African countries have been lagging in implementing the regulatory framework. It is thus up to African countries to take advantage of the multiple initiatives designed to strengthen our legal framework.^16^

 Africa has the highest vaccine demand due to the rise in infectious disease cases. Africa’s existing vaccine market is currently estimated at $1.3 billion and is projected to reach $2.35 billion by 2030. This is a result of population growth and more widespread vaccination due to increased vaccine reception. African countries are graduating from the Global Vaccine Alliance’s low-cost scheme to purchase directly from vaccine manufacturers. However, this might imply the potential of mass purchase of vaccines (large market).^17,18^ Investors see a small vaccine market due to the low purchasing power of the citizens, the same with the highest demand for vaccines due to the rise in infectious diseases cases; there is a potential for mass purchase (large market) seen. Stakeholders’ interests in investing in vaccine development in Africa are also discouraged due to the different political ambitions among the African Government, making market prediction difficult. This call for the need to make the vaccine market lucrative enough to attract major stakeholders and provide policies which would encourage their participation as well as pharmaceutical firms’ interests which would, in turn, trigger vaccine research by attracting sufficient funds required.^17^

 Disease surveillance and data collection are also poor in most African countries. This affects vaccine research as it prevents research institutes from having precise forecasts and numerical statistics of the disease burden and the open market opportunity to trigger research and attract needed support.^19^

## Recommendations

 Africa region needs to take proactive steps and develop precise strategies by looking at possible ways to enhance vaccine development in the African region. Some of the recommendations are highlighted below:

###  Sufficient funds for research through grants and programs

 To ameliorate the problem of insufficient funds, basic research ethics have to be implemented because sufficiently investing in vaccine research will enhance better health outcomes in Africa. An increase in the amount and complexity of health research in sub-Saharan Africa suggests the need for continuous investment in research ethics capacity development. Structural development of partnerships and networks is a way of raising funds for vaccine research in Sub-Saharan Africa. Although support from international findings has hampered African researchers from leading and fostering research for the benefit of Africa effectively, the African Governments should develop sustainable investment schemes for funding vaccine research systems. Ingenious schemes like Medical Education Partnership (MEPI) and Human Heredity and Health Africa have assisted investigators in promoting vaccine research in previous times.^4^ In some African countries, less than US$ 59 is spent on the health sector as the annual per capita expenditure; this is insignificant for health research.^20^

###  Efficient surveillance system

 The accessibility of African researchers to health information such as birth and death registration, census, and disease surveillance improves the effectiveness of executing public health research^4^. Clear numerical statistics and a precise forecast of disease burden in Africa can only be possible when initiatives on the line are identified and well-prioritized. An authoritative source for cancer data in the United States is the Surveillance, Epidemiology, and End Result Program (SEER) (National Cancer Institute) in the same line African Cancer Registry Network,^21^ which has been on implementation in Africa to provide information on disease statistics in an attempt to reduce its burden. Building a disease surveillance program around a population-based registry is pivotal in monitoring and evaluating the burden for further scrutiny and its control.^22^ As there is an evolution in the discovery and occurrence of disease burden, there has to be room for reinforcement of infrastructure and personnel.

 Gathering high-quality population-based incidence and mortality data on all disease burdens for all age groups by cancer type, including measurements of inequalities, through population-based cancer registries, household overviews, and other health information systems to guide policies and plans.^23^ There is a need for demographic health surveillance to be in place as this will help investigators know the number and location of potential or vulnerable individuals. Media outlets should help to share accurate, make public education on safe vaccination as this rule out anti-vaccine sentiments. Community-level health events should be captured and recorded, and the data should be submitted to the appropriate portals. These disease surveillance strategies will clarify the disease burden and the market demand.^24^

###  Proper legislative framework and policy making

 The African countries should get back to the African Vaccine Regulatory Forum (AVAREF) invented in the year 2006, which was the aftermath of the workshop held by the World Health Organization.^16^ Besides, the political will and commitment of African governance must be improved for WHO officials to assist in vaccine research and manufacturing in Africa.^17^

###  Adequate vaccine effectiveness programs

 The effectiveness of vaccination programs in Africa relies majorly on vaccine acceptance. Hence, public health experts in Africa should intensify their efforts in tackling anti-vaccine activists through community and religious leaders’ sensitization and massive social media vaccine enlightenment campaigns targeting the youth.^25^ Local production of vaccines is another way to break mistrust around vaccination programs and to ensure adequate effectiveness of the programs. Therefore, Government and policymakers should channel resources into vaccine development and production, as seen in South Africa and Kenya.^25^

## Conclusion

 Vaccine research and development is essential in any nation, the involvement in vaccine research has been seen to effortlessly defend individuals against irresistible diseases and reduce the burden of these diseases. Most African countries are daunted with challenges in engaging in vaccine research due to a lack of adequate research funding and technological investments; this emphasizes the need to encourage vaccine research in Africa.

 With the hope that African countries will acquire all they need to develop vaccines, it is essential that African countries tackle the challenges of vaccination, ensure they have the proper vaccine storage facilities, and engage in vaccination programs. The policymakers also have an essential role to play. Vaccine research demands significant technological investments, so policymakers should likewise direct resources toward vaccine development.

## Acknowledgements

 We acknowledge Pharm Incubation Hub for the mentorship and guidance provided throughout the writing stage of this manuscript.

## Author Contributions

**Conceptualization:** Yusuff Adebayo Adebisi.

**Project administration: **Adedoyin John-Joy Owolade.

**Resources: **Adedoyin John-Joy Owolade.

 Supervision: Adedoyin John-Joy Owolade & Taiwo Oluwaseun Sokunbi.

**Validation: **Yusuff Adebayo Adebisi.

**Writing original draft: **Adedoyin John-Joy Owolade, Taiwo Oluwaseun Sokunbi, Favour Oluwatobi Aremu, Esther Oluwatosin Omotosho & Blessing Abai Sunday.

**Writing review & editing: **Yusuff Adebayo Adebisi, Aniekan Ekpenyong & Abdulhammed Opeyemi Babatunde.

## Funding

 This research did not receive any specific grant from funding agencies in the public, commercial, or not-for-profit sectors.

## Ethical Approval

 Ethical approval was not required for the manuscript

## Competing Interest

 Authors declare no competing interest.
